# A Plane Stress Failure Criterion for Inorganically-Bound Core Materials

**DOI:** 10.3390/ma14020247

**Published:** 2021-01-06

**Authors:** Philipp Lechner, Christoph Hartmann, Florian Ettemeyer, Wolfram Volk

**Affiliations:** 1 Chair of Metal Forming and Casting, Technical University of Munich, Walther-Meissner-Strasse 4, 85748 Garching, Germany; christoph.hartmann@utg.de (C.H.); wolfram.volk@utg.de (W.V.); 2 Fraunhofer Research Institution for Casting, Composite and Processing Technology IGCV, Walther-Meissner-Strasse 4, 85748 Garching, Germany; Florian.ettemeyer@igcv.fraunhofer.de

**Keywords:** foundry cores, foundry core materials, Mohr-Coulomb, Weibull, fracture strength, water-glass, tri-axial testing, hydrostatic pressure

## Abstract

Inorganically-bound core materials are used in foundries in high quantities. However, there is no validated mechanical failure criterion, which allows performing finite-element calculations on the core geometries, yet. With finite-element simulations, the cores could be optimised for various production processes from robotic core handling to the decoring process after the casting. To identify a failure criterion, we propose testing methods, that enable us to investigate the fracture behaviour of inorganically-bound core materials. These novel testing methods induce multiple bi-axial stress states into the specimens and are developed for cohesive frictional materials in general and for sand cores in particular. This allows validating failure criteria in principal stress space. We found that a Mohr-Coulomb model describes the fracture of inorganic core materials in a plane stress state quite accurately and adapted it to a failure criterion, which combines the Mohr-Coulomb model with the Weakest-Link theory in one consistent mechanical material model. This novel material model has been successfully utilised to predict the fracture force of a Brazilian test. This prediction is based on the stress fields of a finite element method (FEM) calculation.

## 1. Introduction

Inorganically-bound (IOB) core materials are used in large-scale light-metal foundries to form cavities in cast parts, which are not possible with a permanent mould, since they need to be free of undercuts [1]. The cores are lost cores, which are used once and have to be destroyed in order to remove them from the cast part. This process is called decoring [2].

In contrast to organically-bound cores, inorganic core materials do not combust during the casting process, which has ecological benefits [3]. However, this also leads to a higher remaining core strength after the casting process, which increases the necessary effort during the decoring of the cast parts [4].

**Figure 1:**
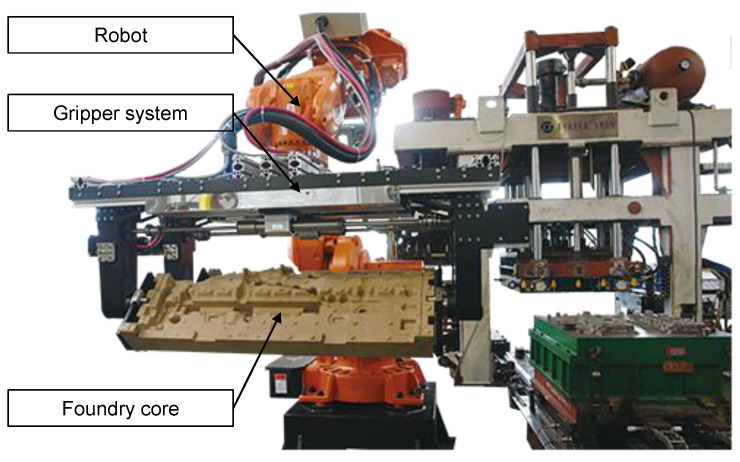
Core handling with a robot [8]. The design of the gripper and the locations of contact with the core determine the stresses in the core.

Due to the quantity of cast parts with inorganically-bound cores, it is desirable to perform FEM (finite element method) optimisation for various mechanical processes, like handling and decoring, which is already state-of-the-art for most materials used in large-scale industrial productions. In previous work, Lechner et al. measured the elastic parameters of inorganic core materials [5]. However, a consistent and validated mechanical material model that allows strength calculations of real geometries with complex stress states is currently not available. From the core shooting with its subsequent mechanical handling processes to the destruction of the cores in the decoring process, applications of a calculation model for inorganic cores are manifold along the life cycle of the cores. One exemplary technical processes, which will benefit from a FEM process optimisation, is the core handling. During the automatic handling, robots and gripper systems are often utilised to transport the foundry cores to the casting mould and to place to cores inside the mould [6]. In most cases, the gripper has to be specifically designed for this purpose. The gripping force and the points of contact to the core have to be chosen. The core has to be safely fixated but must not be damaged [7]. Without a validated material model, this process has to be on a trial and error basis. This may lead to the necessity of redesigning the gripper after the first cores have been produced. With an FEM calculation, this could be avoided and the gripper can be properly designed early in the product development process. Figure 1 shows a typical example of core handling. Due to the force of the gripper and the weight of the product, the stresses in the core are considerable.

This article is structured as follows: After the introduction with the motivation, the state of the art and the theoretical foundations, the materials and methods are described in Section 2. Subsequently, the testing methods are utilised to determine the fracture stress of inorganically-bound core materials for several plane stress states and to fit two yield criteria to the data. In Section 4, a novel formulation of the Mohr-Coulomb criterion is proposed and fitted to the data. Section 5 validates this model, by predicting core fracture under a complex stress state, while Section 6 discusses the results of this article.

### 1.1. Fracture Strength of Inorganically-Bound Core Materials

*Bending* Bending tests are the most commonly used test of fracture strength for core materials. For example Stauder et al. test the properties of foundry sand cores with three-point-bending (3PB) [9]. Griebel et al. used a four-point-bending setup to determine the Young’s modulus of various foundry core materials [10].

*Compression* Similar to bending, the compression test is widely used for foundry core materials. Izdebska-Szanda et al. use the compression test to characterise the strength of foundry core materials [11]. Popoola and Fayomi utilise the compression test to determine green core strength [12], while Brück and Ratke test the performance of an entirely new binder with it [13].

*Bi-axial bending* For ceramics, there are various related bi-axial bending tests in use. They have in common that a plate-shaped specimen is supported by circular positioned balls and are loaded either by one central ball [14] or by a concentric ring [15]. Depending on the number of support balls, the supports are modelled mostly as point-loads [16]. Ramakrishnan et al. adapted the ball-on-three-balls test for IOB core materials [17].

### 1.2. Failure Criteria

There are various material models from engineering and geo-sciences, which, in theory, could be applied to IOB core materials. This assumption is based on the similar porous structure, which is built from physically or chemically-bound particles. This class of materials is also called cohesive frictional materials [18]. For these models, the model complexity directly correlates to the number of parameters utilised to describe to failure surface. The hydrostatic stress is an important influence on the fracture stress of frictional materials [19]. Failure criteria and yield loci with only one parameter, like the von Mises [20] and the Tresca [21] criterion, which neglect the hydrostatic stress, should not be utilised. Therefore, two parameter models, like the Mohr-Coulomb [22] and the Drucker-Prager [23] criterion, represent the minimum model complexity. The Drucker-Prager criterion is a promising model for IOB core materials, since we assume an influence of the hydro-static pressure on the fracture strength, due to similarities to the porous materials in geo-sciences. Furthermore, Galles and Beckermann model sand cores in steel casting with a Drucker-Prager criterion [24]. Thorborg et al. also utilise a Drucker-Prager model for core materials [25]. The Mohr-Coulomb failure criterion is often used in geo-sciences as well, since soil is known to fail in shear bands [18]. Stauder et al. model foundry cores with the Mohr-Coulomb criterion [26]; however, there is no full validation.

The general shape the Drucker-Prager and the Mohr-Coulomb model have inspired a multitude of more complex failure models for frictional materials. Two examples are the unified failure criterion of Mengcheng et al. [27] and the Ehlers criterion [18]. Moreover, there is research regarding the failure of concrete, which could be of interest for inorganic core materials. Menetrey and Willam proposed a three parameter model, which describes the failure of concrete [28]. Červenka and Papanikolaou adapted a Rankine criterion to concrete [29], while Sucharda utilised a fracture-plastic material model for concrete [30].

However, in this article, we focus on the Mohr-Coulomb and the Drucker-Prager model, since the small number of parameters offers the chance to parameterise them with two well-chosen experiments. If the need for a more complex material description arises, we will try to adapt these models, such that no further experimental effort is necessary to parameterise the criterion. With regard to an industrial application, it is important to built on the experimental capabilities in typical sand-core laboratories, which are bending [9] and in part compression experiments.

#### 1.2.1. Drucker-Prager Failure Criterion

**Figure 2:**
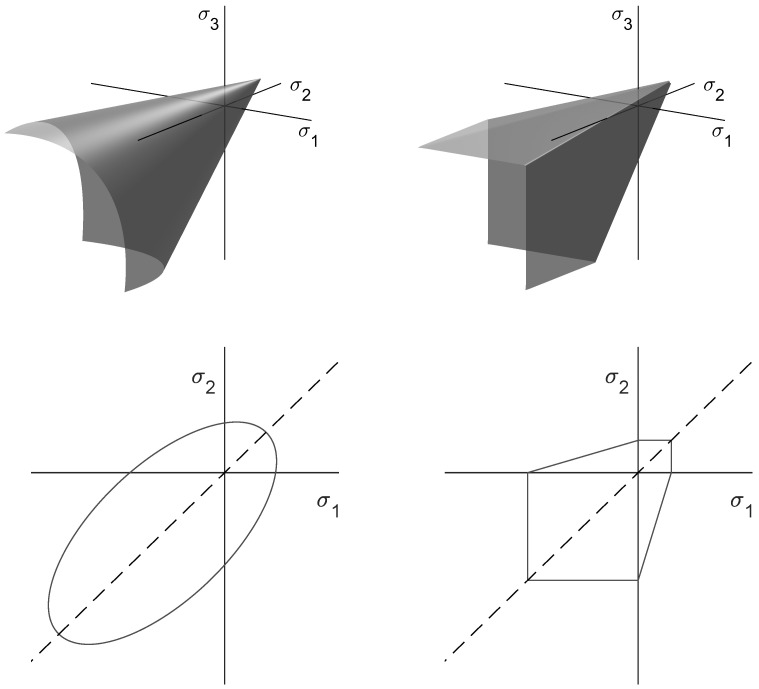
Failure criteria Drucker-Prager (**left**) and Mohr-Coulomb (**right**) in principal stress space. The upper half describes three-dimensional stress states, while the lower half describes plane stress states ($\sigma_{3}=0$).

The Drucker-Prager failure criterion is mainly used for problems of soil and concrete mechanics. In three-dimensional principal stress space, its failure surface has the shape of a cylindrical cone along the hydrostatic axis ($\sigma_{1}=\sigma_{2}=\sigma_{3}$). The following equation describes the Drucker-Prager failure surface, which is shown in Figure 2 (left) [23]:

(1) $$\alpha I_{1}+J_{2}^{1/2}-k=0,$$

(2) $$I_{1}=\sigma_{1}+\sigma_{2}+\sigma_{3},$$

(3) $$J_{2}=\frac{1}{6}\left[{\left(\sigma_{1}-\sigma_{2}\right)}^{2}+{\left(\sigma_{2}-\sigma_{3}\right)}^{2}+{\left(\sigma_{3}-\sigma_{1}\right)}^{2}\right],$$

where $I_{1}$ is the first invariant of the stress tensor, and $J_{2}$ is second invariant of the deviatoric stress tensor. $\sigma_{1}$, $\sigma_{2}$, and $\sigma_{3}$ are the principal stresses. α and *k* are the parameters describing the shape of the cone.

#### 1.2.2. Mohr-Coulomb Failure Criterion

The Mohr-Coulomb failure criterion describes shear-induced failure. Similar to the Drucker-Prager criterion, it is a two-parameter model, which has the shape of a hexagonal pyramid along the hydrostatic axis. The associated failure surface is shown in Figure 2 (right) and described by the following equations [22]:

(4) $$\tau_{max}=c+\sigma_{n}\tan\left(-\phi \right),$$

where $\tau_{max}$ is the shear stress in the failure plane, $\sigma_{n}$ is the normal stress acting on the respective failure plane, *c* is the cohesion of the material, and ϕ is the friction angle. In principal stress space, this translates to a material failure if one of the following conditions is met:

(5) $$\frac{|\sigma_{1}-\sigma_{2}|}{2}=\frac{\sigma_{1}+\sigma_{2}}{2}\sin\left(-\phi \right)+c\;\cdot \;\cos\left(-\phi \right),$$

(6) $$\frac{|\sigma_{2}-\sigma_{3}|}{2}=\frac{\sigma_{2}+\sigma_{3}}{2}\sin\left(-\phi \right)+c\;\cdot \;\cos\left(-\phi \right),$$

(7) $$\frac{|\sigma_{3}-\sigma_{1}|}{2}=\frac{\sigma_{3}+\sigma_{1}}{2}\sin\left(-\phi \right)+c\;\cdot \;\cos\left(-\phi \right).$$

## 2. Materials and Methods

In the following, we describe various test setups to determine fracture strength of IOB core materials. All tests are performed on a universal testing machine ‘Z020’ equipped with a 20 kN force sensor (ZwickRoell GmbH & Co. KG, Ulm, Germany). Please note that the fracture of IOB cores is following a Weibull distribution. This makes a correction of the fracture stresses according to the effective volumes for each test setup necessary [31]. Comparing specimens with a shape factor *m* and different volumes *V*, the fracture stress $\sigma_{s}$ follows:

(8) $$\frac{V_{1}}{V_{2}}={\left(\frac{\sigma_{s_{2}}}{\sigma_{s_{1}}}\right)}^{m}.$$

The effective volume for each test setup can be calculated such that:

(9) $$V_{eff}=\int_{V}{\left(\frac{\sigma }{\sigma_{max}}\right)}^{m}dV,$$

where σ is the local stress in the volume cell used for the integration, and $\sigma_{max}$ is the maximum stress in the specimen.

### 2.1. Specimens

**Figure 3:**
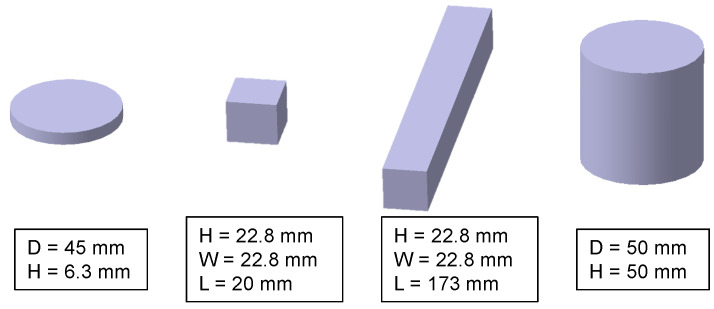
Specimens used in this study: A disc with 45 mm diameter and 6.3 mm height, two cuboids with a 22.8 × 22.8 mm^2^ cross-section and a length of 20 mm and 175 mm, respectively, and a cylinder with 50 mm diameter and 50 mm height.

There are three types of specimens produced for this study, which are shown in Figure 3. A bending beam with a quadratic cross-section with 22.8 mm edge length and a length of 175 mm, a disc with 50 mm diameter and 6 mm width, and a cylinder with 50 mm diameter and 50 mm height. The bending beam is either used for bending experiments in its full length or shortened to a cubical shape and a length of 20 mm for compression tests. The disc is used for bi-axial bending tests. Both tests will be described in the following section.

**Figure 4:**
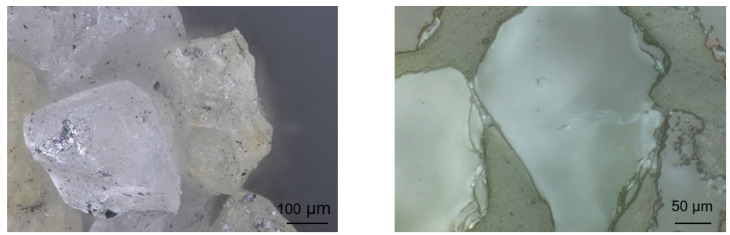
Microscopic analysis of the sand-binder micro-structure. On the left-hand side, an unprocessed sand-binder structure is depicted, while it is polished on the right side.

Consistent with previous studies, the specimens were produced on a Loramendi SLC2 25L core-shooting machine (Loramendi S.Coop., Vitoria, Spain) with a heated tool and a hot-gassing device. A H32 silica sand (Quarzwerke GmbH, Frechen, Germany) with an average grain size of 0.32 mm was bound with an inorganic Inotec binder system (ASK Chemicals GmbH, Hilden, Germany). The sodium silicate binder system consists of a liquid component EP 4158 (2 wt%) and a powder additive TC 4500 (1.6 wt%), which are measured relative to the sand mass. The temperatures were chosen at 155 °C core box and 220 °C hot-gassing temperature. Figure 4 shows a microscopic analysis of the sand-binder micro-structure. The hardening of the binder results in binder bridges between the individual sand grains. The specimens were stored at 20 °C and 10% humidity after production [31].

### 2.2. Uni-Axial Compression Experiment

We used the 20 mm × 22.8 mm × 22.8 mm cubic for uni-axial compression tests. The load *F* was induced on the 22.8 mm × 20 mm surface area *A* by the testing machine. Thus, the fracture stress was calculated to:

(10) $$\sigma_{p}=\frac{F}{A}.$$

**Figure 5:**
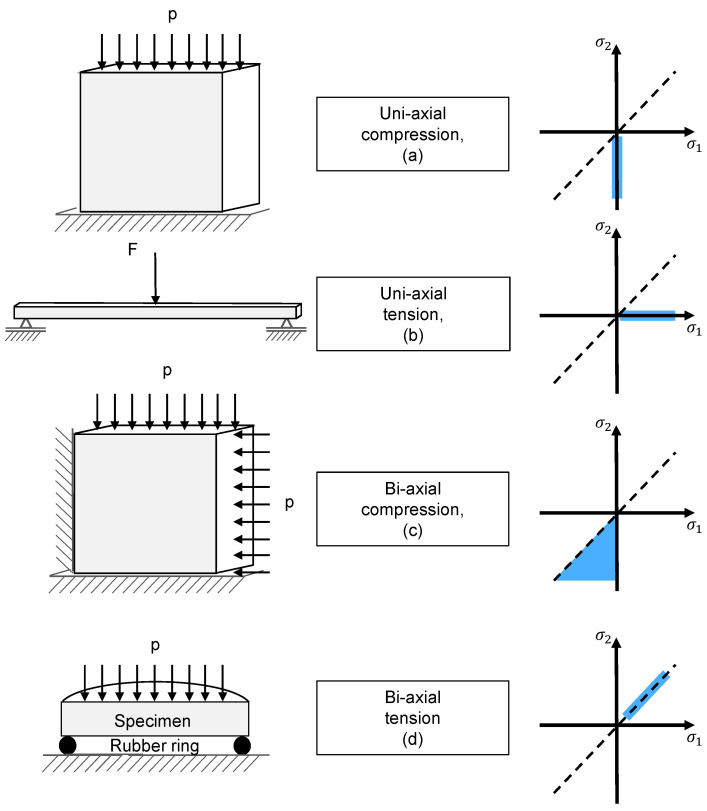
Uni- and bi-axial testing methods for compression (**a**), tensile (**b**), bi-axial compression (**c**), and bi-axial tensile (**d**), together with their respective position in principal stress space.

The position of this and the following experiments in principle stress space are shown in Figure 5.

### 2.3. Uni-Axial Tension Experiment

We choose the three-point-bending experiment to measure uni-axial tension fracture stresses, since the results are experimentally more stable than other known methods to induce uni-axial tension, like the dog-bone and the Brazilian disc test. The dog-bone test is unstable regarding the clamping of the specimen to the testing machine, since a lot of specimens fracture in the vicinity of the clamping mechanism, which renders the result invalid. The Brazilian disc test induces pure tension into the specimen in theory, but, studies have shown that, in reality, there is a multi-axial complex stress state around the two load points [32]. This excludes the Brazilian disc test, since we are searching for experiments with stress states as simple and stable as possible, both for uni- and bi-axial stress, to validate a material model for IOB core materials.

We used the bending beam described in Section 2.1 with a distance *c* of 150 mm between the supports and a force *F*. The calculation of the maximum fracture stress in the beam follows Bernoulli’s beam theory [33]:

(11) $$\sigma_{3PB}\left(x,z\right)=\frac{\frac{F_{3PB}}{2}\left(x-2{\langle x-\frac{c}{2}\rangle }^{1}\right)}{I}z,$$

where $\sigma_{3PB}$ is the stress in the beam at the coordinates *x* and *z*. *x* is the coordinate along the beam’s length, while *z* is the distance from the neutral layer. $F_{3PB}$ is the maximum force at fracture, and *I* is the geometrical moment of inertia.

### 2.4. Bi-Axial Compression Experiment

For the bi-axial compression stress state, we developed a test jig, which allows introducing compression stresses into the cubic specimen. The test jig consists of a support and two triangular wedges. The support has three planar surfaces, which are perpendicular and act as mechanical stops on three of the six sides of the cube. These surfaces position the cube in the support. On two of the free sides are inclined planes with an angle of 60°. This allows inserting wedges into the test setup, which redirect the force of the testing machine onto two sides of the specimen. The force is induced into the specimen via separate plates to avoid a friction contact between the moving wedge and the specimen. The wedges are coated with Polytetrafluorethylen (PTFE) to reduce friction in the test setup. The force of the testing machine is applied to the wedges via two force sensors S20S (Bosche GmbH & Co. KG, Damme, Germany). The contact between the wedges and the force sensor can be adjusted with a screw, which allows for asymmetrical stress states in the specimen.

**Figure 6:**
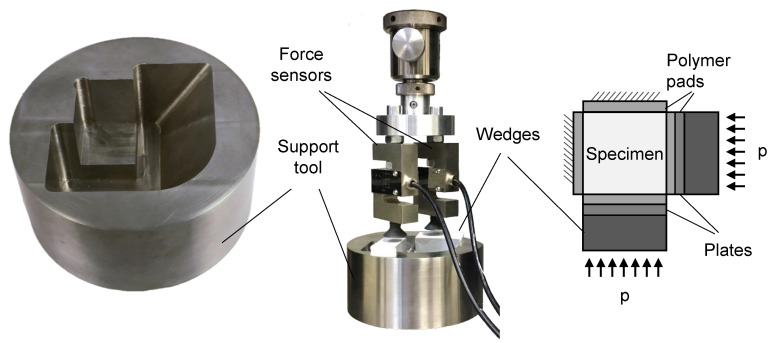
Test setup for bi-axial compression experiments on a universal testing machine.

All surfaces with a contact to the specimen are equipped with a 1 mm polymer pad to ensure an evenly distributed stress field and to allow for approximately free transversal contraction of the specimen. The last side of the cube remains free to enable for an approximate plane stress state when neglecting the stresses resulting from transversal contraction and friction. An outline of the test jig and its flow of forces is shown in Figure 6. With the angle of the wedges, the force $F_{s}$ applied to one side of the specimen can be calculated to:

(12) $$F_{s}=\tan\left(\pi /3\right)F_{p},$$

with $F_{p}$ being the force of the testing machine on each wedge. We quantified the friction in comparison to a classical compression experiment. If only one of the two wedges is applied in the test setup, a uni-axial compression state is achieved, which can be directly compared to the standard uni-axial compression test. We found that the fracture stress in the new test setup is increased by 7.4%, due to the friction of the moving wedges. This friction factor can be applied to both wedges for reasons of symmetry. In the following, this friction is subtracted from the test results.

### 2.5. Bi-Axial Tension Experiment

Bi-axial tension experiments are often used for brittle materials, like ceramics. As mentioned in Section 1.2, the tests most often used are the ball-on-ring and the ball-on-three balls experiment. However, both tests are inadequate to measure Weibull material parameters for IOB core materials, since both are based on point contact. While this is acceptable for hard materials, like ceramics, with IOB core materials, the surface of the specimen is easily damaged, which transforms the near-perfect point contact to a groove. This changes the stress state considerably compared to the respective mechanical model based on plate theory.

**Figure 7:**
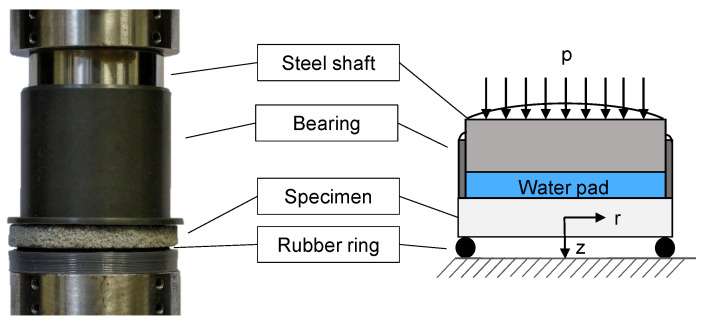
Bi-axial bending test setup. A circular disc specimen is placed on a rubber ring and loaded with a surface pressure through a water-filled pad. The resulting bi-axial stresses can be calculated with plate theory.

To avoid this problem, we designed an experiment for bi-axial tension stress states based on the Bulge experiment [34], which is shown in Figure 7. A plate-shaped specimen is placed on a ring for support. In case of IOB core materials, a flexible polymer ring is used, since it distributes the load of the support more evenly onto the specimen, even if the surface is not perfectly flat. A shaft bearing is placed upon the specimen, whose inner diameter is equal to the diameter of the support ring. We placed a polymer balloon, which is filled with water, inside this bearing. The force of the universal testing machine is applied to this water pad through a piece of shaft. Due to the constraints of the bearing and the shaft on the water pad, the force is evenly distributed over the area of the specimen. The test setup is shown in Figure 5e. The bi-axial stress state can be described with [35]:

(13) $$\sigma_{r}=\frac{3pa^{2}}{4}\left(3+\nu \right)\left(1-\frac{r^{2}}{a^{2}}\right)\frac{z}{b^{3}},$$

(14) $$\sigma_{\phi }=\frac{3pa^{2}}{4}\left[2\left(1-\nu \right)+\left(1+3\nu \right)\left(1-\frac{r^{2}}{a^{2}}\right)\right]\frac{z}{b^{3}},$$

where $\sigma_{r}$ and $\sigma_{\phi }$ are the radial and the tangential stresses, respectively. *p* is the applied pressure, and *a* and *b* are the radius and the width of the specimen. ν is the Poisson ratio. *r* and *z* are the two variables, which describe the location on the specimen (radius and distance from the neutral plane). Assuming that shear stresses are negligible, $\sigma_{r}$ and $\sigma_{\phi }$ are equal to the principal stresses $\sigma_{1}$ and $\sigma_{2}$. Lechner et al. found that the Poisson ratio for the core material, used in this article, is approximately 0.18 [5]. This Poisson ratio is used in the following, as well.

### 2.6. Validation Experiment

**Figure 8:**
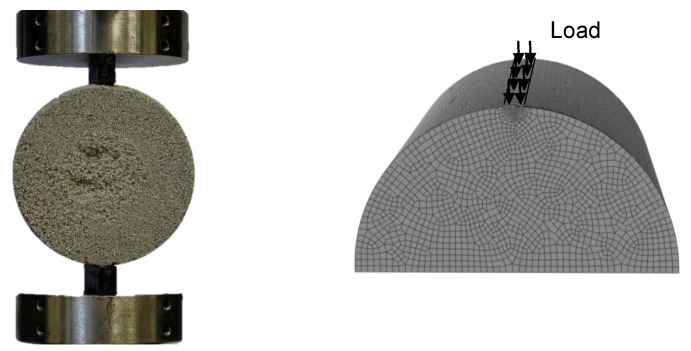
Brazilian test setup. To accurately describe the contact surface the Brazilian test was adapted with polymer supports with a circular contact surface (**left**). This load was modelled with a surface pressure in a symmetrical finite element model (FEM) model (**right**).Brazilian test setup

The multi-axial and complex stress state, which is induced by the Brazilian test is well suited for a validation experiment after the material characterisation. We will perform experiments and a FEM simulation and compare the prediction of the fracture force with the experimental results to validate the utilised material models. The classic Brazilian test setup induces a diametrical load into the cylindrical specimen with two parallel plates. In theory, the contacts between the specimen and the testing machine are line contacts. Due to the elasticity of the material the line contact becomes a small contact surface with high pressure. This type of contact is difficult to model in a simulation, which leads an adaption of the Brazilian test. We utilised two polymer parts with 5 mm width and 50 mm length to transmit the load to the specimen. The parts are rounded in the contact area with a radius of 25 mm, which fits to the radius of the specimen. This leads to a well defined contact between the test setup and the specimen. The surface pressure can be approximated with a homogeneous stress, which is calculated with the load force and the contact area. This test setup is shown in Figure 8 (left). The specimen is modelled with one symmetry in the FEM software Abaqus 2018. The load is induced with a surface pressure boundary condition on the area of contact. The model is meshed with cubic elements of type C3D8R with an edge length of 2 mm in general and an edge length of 0.5 mm in the vicinity of the contacts. In total 156,100 elements are utilised. The model is shown in Figure 8 (right). The simulation is parameterised with a Young’s modulus of 5.75 GPa and a Poisson ratio of 0.18, which was acquired in a previous publication [5]. The density of the material was determined to 1570 kg/m3. The calculated stress tensors and element volumes are exported to Matlab. Subsequently, the effective volume is calculated from the stress tensors and the element volumes according to Equation (9). Which yield criterion is suitable best will be decided according to the following experiments. However, the strength parameter of the utilised yield criterion is scaled to the effective volume of the Brazilian test with Equation (8), according to Weibull’s theory. Finally, the stress fields are evaluated for material failure according to the yield criterion in question.

## 3. Experimental Results

Please note that in this section all experimental results are scaled to the effective volume of the three-point-bending experiment according to Weibullian statistics and the approach proposed by Lechner et al. in an earlier article [31]. Furthermore, we only determined data points on one side of the hydrostatic axis in Figure 2, due to the axial symmetry of the Drucker-Prager and the Mohr-Coulomb criteria.

### 3.1. Uni-Axial Results

**Figure 9:**
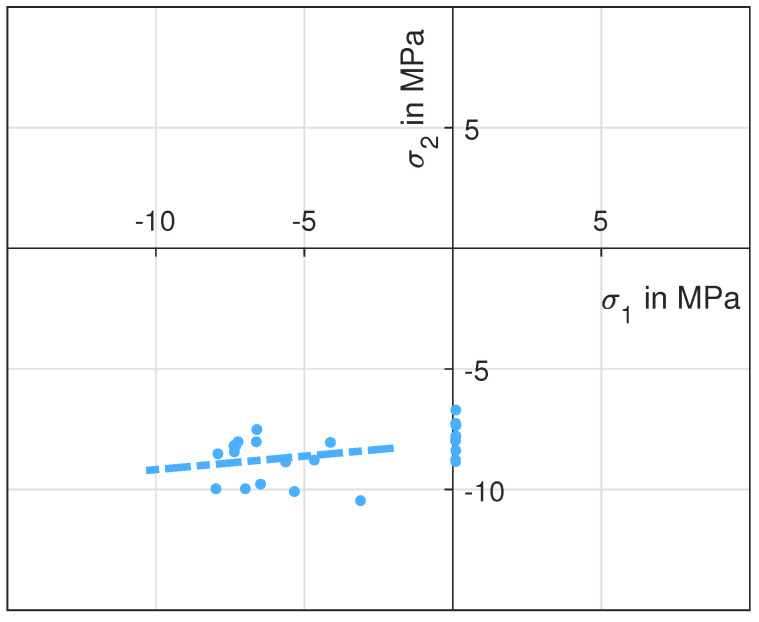
Test results of the uni- and bi-axial compression tests. The dashed line is a linear best fit to the test results.

**Figure 10:**
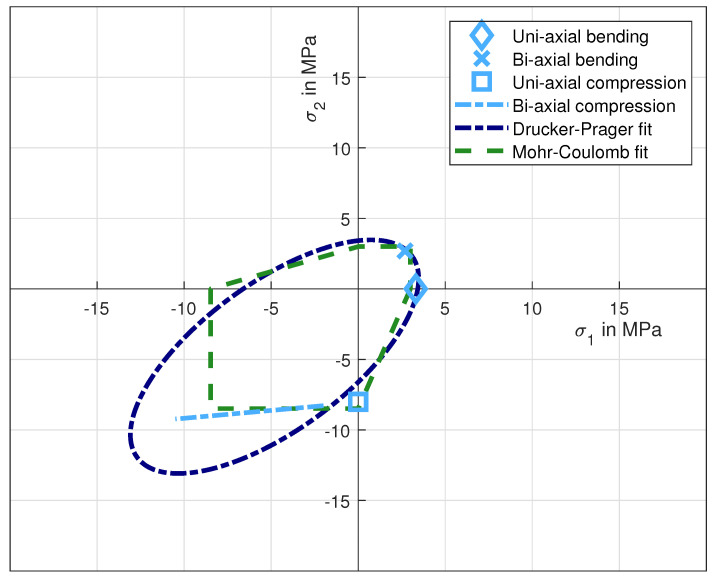
Experimental results for strength of inorganically-bound (IOB) core materials loaded with various uni- and bi-axial stress states. A Drucker-Prager and a Mohr-Coulomb model are fitted optimally to the test results.

To determine the failure strength for uni-axial stress states, we utilised three-point-bending and compression test experiments. The bending stress was calculated with Equation (11) to a Weibull scale parameter of 3.3 MPa with a shape parameter of 36 (8 specimens). The compression strength was determined to be 8.01 MPa with a shape parameter of 11 (10 specimens). The single compression test results are shown in Figure 9, and the Weibull data points for both uni-axial test setups are depicted in Figure 10 as a diamond and a square, respectively.

**Figure 11:**
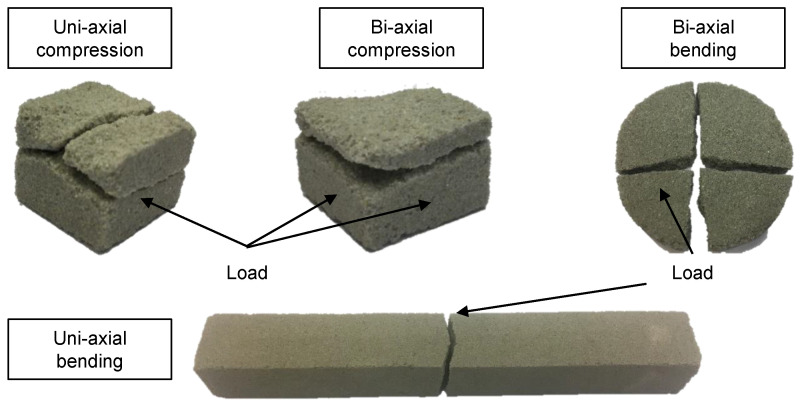
Typical examples of fractured specimens for the performed experiments. The compression and uni-axial experiments show signs of a shear failure.

Typical examples of fractured specimens are shown in Figure 11. The black arrows mark the sides of the specimens, which are utilised to induce the load. The crack path indicates shear failure, especially in the uni-axial compression experiment. The crack path of the uni-axial bending experiment is probably determined by the local micro-structure and the path from the tensile stress maximum to the compressive stress maximum with the lowest local material strength.

### 3.2. Bi-Axial Compression Experiment

The results of the bi-axial compression experiments are shown in the compression-compression quadrant of Figure 9. The test results (23 specimens) are a point cloud along the $\sigma_{1}$ axis, since it is not possible to distribute the force of the testing machine perfectly symmetrical to the two wedges. The distance between the wedges and the punch can be adjusted by screws. However, the position of the wedges is defined by the exact dimension of the specimen, which is underlying typical tolerances. We adjusted the screws such that most points are close to the axis of symmetry, since this area distinguishes the Mohr-Coulomb criterion from the Drucker-Prager criterion very well. Furthermore, we also adjusted $\sigma_{1}$ such that a uni-axial stress state was achieved. We did not calculate one data point for the compression-compression quadrant. Instead, we fitted a linear function into the point cloud including the uni-axial test results, as shown by a dashed line in Figure 9. In our opinion, this line describes the mechanical failure behaviour in this quadrant better than a single data point and the slope of this line will discriminate between the Mohr-Coulomb and the Drucker-Prager criterion. The $\sigma_{2}$ fracture stress is nearly constant with a small increase towards higher values of $\sigma_{1}$. With the uni-axial compression results from the standard compression test and from this test setup, the friction in the bi-axial test setup can be quantified to 7.4%. The scale parameter and the mean value of the $\sigma_{2}$ component can be found in Table 1, as well. The course of the crack is similar to the uni-axial compression experiment and shows signs of a shear failure. However, the bi-axial load leads to a crack, which results in only two parts. Comparing the uni-axial and bi-axial crack indicates that it originates on both loaded surfaces for the bi-axial compression experiment. The cracks meet in the specimen, which leads to the two remaining parts.

### 3.3. Bi-Axial Tension Results

**Figure 12:**
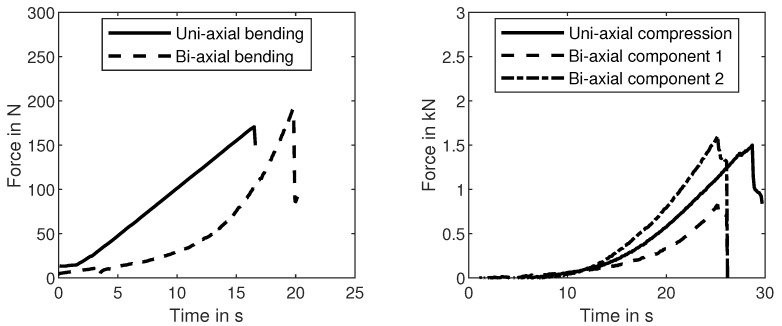
Typical examples of force signals of the performed experiments. On the left-hand side, one example for each of the bending experiments is depicted, while the two compression experiments are shown on the right. The bi-axial compression experiment has two independent force components.

We determined the bi-axial tensile strength with the new bulge-like experiment described in Section 2.5. The radial and tangential stresses were calculated with Equations (13) and (14). Assuming that shear stresses are negligible in this case, these stresses are equivalent to the principal stresses in the disc. The maximum tensile stresses are expected in the middle on the lower surface of the disc. The principal stresses are equal in the middle of the disc, due to the point symmetry of the whole test-setup. After scaling the results to the effective volume of the three-point-bending experiments, the bi-axial tensile strength was determined to be 2.69 MPa with a shape parameter of 19 (12 specimens). The data point is shown in Figure 10 with the x-symbol. Figure 11 shows that the plate fractures typically into four parts. An example of the raw data for each of the performed experiments is depicted in Figure 12. The results, including a statistical evaluation for each experiment, can be found in Table 1. In Table 1, the mean value is without volume scaling, while the scale parameter is calculated for the effective volume of the three-point-bending experiment.

### 3.4. Optimisation of Model Parameters

We combined these experimental results in two-dimensional principal stress space in Figure 10. Additionally, we fitted a Drucker-Prager and a Mohr-Coulomb model to these experimental results. Both models are depicted in Figure 10 as a dotted and a dashed line, respectively. Clearly, the Mohr-Coulomb model fits better to the experimental data sets than the Drucker-Prager model. In particular, the near constant $\sigma_{2}$ in the compression-compression quadrant cannot be modelled accurately by the Drucker-Prager failure criterion. The Mohr-Coulomb parameters after least-square optimisation are c=2.87 and ϕ=0.61.

## 4. Mohr-Coulomb Failure Criterion Based on Weakest-Link Theory

With the experimental results in this article and previous literature [31], which show that IOB cores follow Weibull statistics and a Mohr-Coulomb failure criterion, we have to answer the question of how both findings can be merged to one consistent mechanical model.

The simplest way to combine Weakest-Link-Theory with the Mohr-Coulomb model is to utilise the principle of independent action (PIA), which states that the probabilities of survival resulting from each of the principal stresses can be multiplied to one probability [36]. Since the Mohr-Coulomb model is based on three shear stresses, we adapted that principle to multiply the probabilities of survival *p* of each shear stress component. The three probabilities can be expressed by adapting the Weibullian probability of survival [37]:

(15) $$p_{i}=\exp\left(-{\left(\frac{\tau_{i}}{\tau_{MC_{i}}}\right)}^{m}\right),$$

where $p_{i}$ is the probability of survival of each of the principal shear stresses $\tau_{i}$ and m is the Weibull shape parameter. $\tau_{mc_{i}}$ is the critical shear stress from the failure criterion described in Equations (5) to (7) (right side of the equations). According to PIA, we multiply these probabilities of survival and calculate a Weibullian scale parameter, which describes the material strength for general complex stress states:

(16) $$1-\prod_{i=1}^{3}p_{i}=1-\frac{1}{\exp1}=0.6321;$$

with Equations (5) to (7) and (15), this leads to:

(17) $$\begin{array}{c} \begin{array}{cc} \exp[ & -{\left(\frac{0.5|\sigma_{1}-\sigma_{2}|}{0.5\left(\sigma_{1}+\sigma_{2}\right)\;\cdot \;\sin\left(-\phi \right)+c\;\cdot \;\cos\left(-\phi \right)}\right)}^{m}\\ \; & -{\left(\frac{0.5|\sigma_{2}-\sigma_{3}|}{0.5\left(\sigma_{2}+\sigma_{3}\right)\;\cdot \;\sin\left(-\phi \right)+c\;\cdot \;\cos\left(-\phi \right)}\right)}^{m}\\ \; & -{\left(\frac{0.5|\sigma_{3}-\sigma_{1}|}{0.5\left(\sigma_{3}+\sigma_{1}\right)\;\cdot \;\sin\left(-\phi \right)+c\;\cdot \;\cos\left(-\phi \right)}\right)}^{m}]\\ = & \exp1^{-1} \end{array}. \end{array}$$

**Figure 13:**
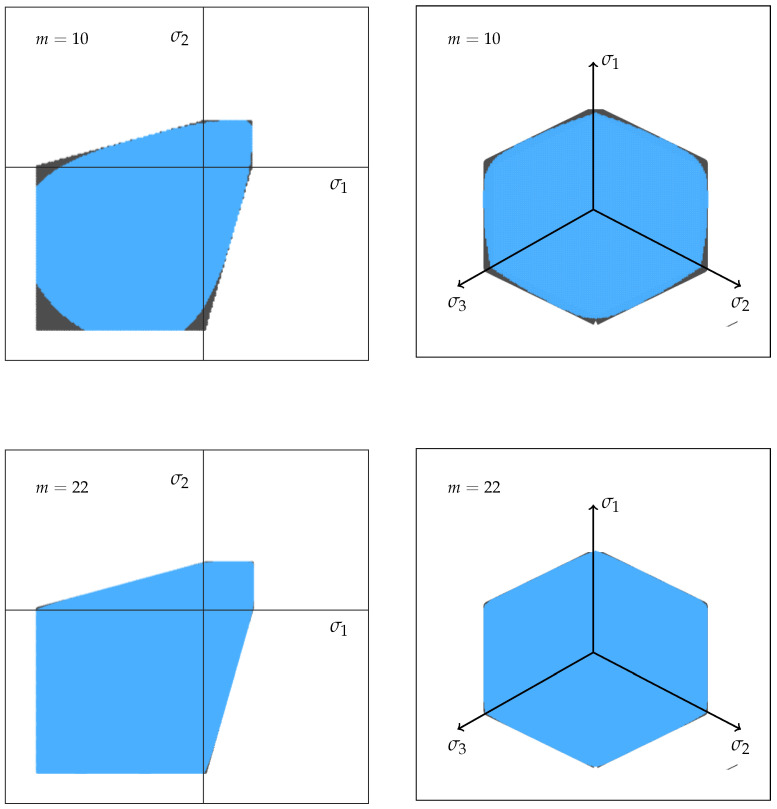
Comparison of the failure surfaces for the theoretical Mohr-Coulomb criterion without scatter (black) and a Mohr-Coulomb criterion adapted with Weakest-Link-Theory (blue). Weibull shape parameters are m=10 and m=22.

This equation represents a three parameter failure criterion for brittle materials based on the Mohr-Coulomb model. Figure 13 shows the result for the shape parameter m=10 (compression experiments) and *m* = 22 (mean of all experiments) for plane stress and in the deviatoric plane. The originally sharp edges of the Mohr-Coulomb model are rounded, since for near critical multi-axial stress states the two smaller shear stresses are contributing significantly to the total fracture probability. This is in contrast to the original Mohr-Coulomb criterion, which only considers the highest shear stress for the fracture. The effect is increasing with an increasing scatter, which corresponds to a lower shape parameter. In case of the compression experiments, this can explain the lower uni-axial strength, compared to the asymmetrical bi-axial compression strength ($\sigma_{1}<\sigma_{2}$). The symmetrical bi-axial compression strength should also be lower than the asymmetrical strength; however, we do not have enough data points in the vicinity of the axis of symmetry to show this effect. The bi-axial bending strength is slightly lower than the uni-axial strength as well. The average shape parameter for all experiments is m=21.8. The optimisation of this new Mohr-Coulomb equation to the experimental data leads to c=2.93 and ϕ=0.61. While the friction angle ϕ remains unchanged, the cohesion *c* is slightly increased compared to the classical Mohr-Coulomb criterion (c=2.87).

Furthermore, we propose to calculate the effective volume of complex core geometries based on the local shear stresses, instead of the maximum principle stresses. In the state of the art, the effective volume is calculated with the assumption of a principle stress criterion. However, we showed in this article, that core materials follow a Mohr-Coulomb criterion. Therefore, the effective volume should be calculated in accordance with the material failure, which is shear-induced. Furthermore, all shear stresses should be taken into account, since they all contribute to the fracture probability. This leads to the following equation to calculate the effective volume:

(18) $$V_{eff}=\int_{V}{\left(\frac{\tau_{1}}{\tau_{max}}\right)}^{m}dV+\int_{V}{\left(\frac{\tau_{2}}{\tau_{max}}\right)}^{m}dV+\int_{V}{\left(\frac{\tau_{3}}{\tau_{max}}\right)}^{m}dV,$$

where $\tau_{x}$ are the principal shear stresses, and $\tau_{max}$ is the maximum shear stress in the specimen. The calculation of the effective volume should reflect the applied failure criterion. The calculation of the effective volume based on the principle stresses makes sense for ceramics, which are often modelled with the Rankine criterion. However, for core materials, a shear-based calculation of the effective volume is better suited.

## 5. Validation

To validate this material model, we performed Brazilian tests as described in Section 2.6. Additionally, the test was simulated with FEM to predict the fracture force and to compare the results. The calculated stress field is evaluated for fracture with the novel weakest-link-based Mohr-Coulomb model and two typical failure criteria from literature as a reference. The first is the classic Mohr-Coulomb criterion without volume scaling, which has been proposed for core materials by Stauder et al. [26]. The second is the classic Drucker-Prager criterion, which is utilised for core materials by Thorborg et al. [25]. The calculated stresses are inserted into Equations (1), (5)–(7), and (17). If the maximum stresses are exceeding the predicted strength, material failure is assumed.

**Figure 14:**
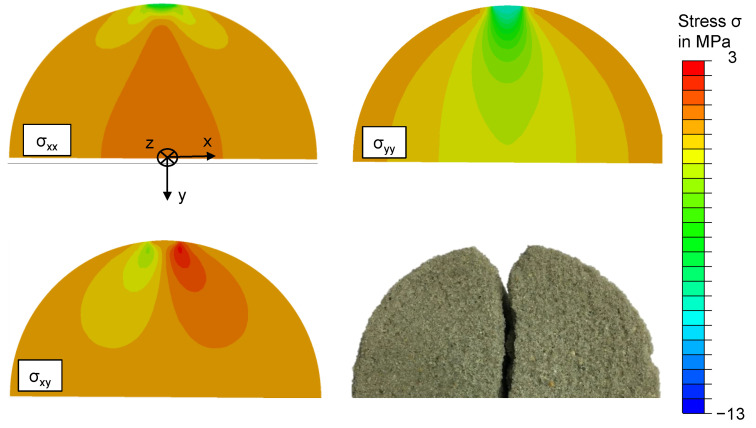
Stress field in the specimen of the Brazilian test. The stress components are calculated with FEM for a load force of 2 kN. The course of fracture in the specimen reflects the stress peaks in these stress components.

Figure 14 shows the stress field in the specimen for a load of 2000 N. Of the nine components of the Cauchy stress tensor, only four show significant stresses, which are compared to one half of a fractured specimen in the figure. The components $\sigma_{xx}$ and $\sigma_{yy}$ are depicted in the upper row, while the component $\sigma_{xy}$ is in the lower row. Since the $\sigma_{xy}$ and the $\sigma_{yx}$ components are symmetrical, only one is necessary. The compressive stresses in the $\sigma_{yy}$ component are significantly higher than the tensile stresses in the $\sigma_{xx}$ component. This validates the assumption that the Brazilian test is not suitable as an experiment for tensile fracture strength, as often done in literature. Furthermore, there is a considerable shear stress component $\sigma_{xy}$. The course of the fracture in the specimen reflects these three stress fields. A triangular gap is generated by two symmetrical stress peaks in $\sigma_{xy}$ near the load points. Subsequently, the course of the fracture is along the stress maxima in the $\sigma_{xx}$ and the $\sigma_{yy}$ components.

**Figure 15:**
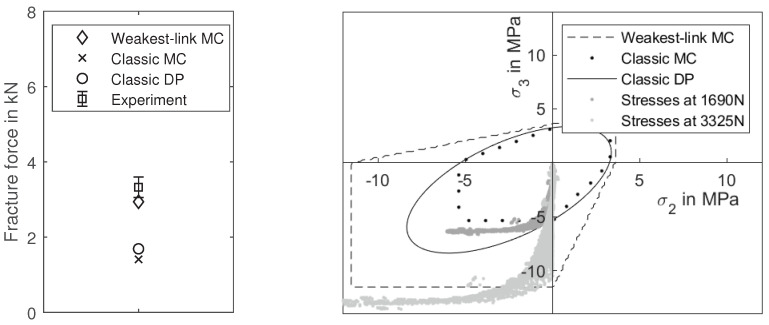
Two major principle stress components in the brazilian test. Each point represents the two major principle stress components at a specific load level. Points in dark grey depict the stress state at a load force of 1690 N, which is the fracture force according to the classic Drucker-Prager criterion. Light grey represents the load level for the experimental fracture force.

For the core material utilised in this publication, the experiments show a fracture force of 3325 N for a sample size of 5 experiments. The classic Drucker-Prager and Mohr-Coulomb criteria are significantly underestimating the fracture force and predict it at 1690 N and 1410 N, respectively. The weakest-link-based Mohr-Coulomb model predicts the fracture significantly better at 2940 N. Figure 15 shows the two major principle stress components for each element in the calculation for two load levels. Dark grey corresponds with the force at which the classic Drucker-Prager model predicts the fracture. Light grey corresponds with the fracture load force in the experiment. It becomes clear that both classic failure criteria are significantly underestimating the fracture force. The Drucker-Prager criterion is indicating material failure in elements with a near uni-axial compression, due to its oval form, while the Mohr-Coulomb model indicates failure in elements with a bi-axial stress field.

## 6. Discussion

Comparing the Drucker-Prager and the Mohr-Coulomb failure criterion to the results of Section 3, the Mohr-Coulomb model describes the fracture behaviour better than the Drucker-Prager model. However, the available data points in the plane stress state emphasise the difference in quality of the fits, since the Drucker-Prager criterion is not able to describe accurately the fracture behaviour in the compression-compression quadrant. The Mohr-Coulomb-based failure criterion for brittle materials, proposed in this article, is rounding the edges of the Mohr-Coulomb criterion depending on the scatter in the experimental data, which brings it closer to the shape of the Drucker-Prager. For the shape parameters, which we extracted from the experiments in this article, the influence is only significant for the compression tests. In any case, it is important to have a consistent mechanical theory, which combines the results of the fracture statistics with the results regarding the macroscopic failure criterion. Furthermore, Equation (16) offers more advantages compared to the classical Mohr-Coulomb Equations (5) to (7). It can be expressed as one implicit equation and the originally sharp edges are rounded, which is advantageous for numerical differentiation. The third parameter *m* does not increase the parameterisation effort, since the scatter can be calculated from the experimental data, which is already necessary for a two-parameter Mohr-Coulomb failure criterion.

Furthermore, we were able to validate the proposed failure criterion for complex stress states in a Brazilian test. Compared to the predictions of a classic Mohr-Coulomb or Drucker-Prager model it predicts the fracture force significantly better. The reason for this difference is the scaling of the material parameters according to the effective volume in the core. The effective volume of the Brazilian test is smaller than the effective volume of the test specimens. Therefore, the fracture force is scaled up, which is evidently necessary to predict the experimental results correctly. This validation experiment further shows that it is possible to predict the fracture load of arbitrary load cases in simulation with the methodology proposed in this publication. This knowledge can be directly applied to handling simulations of foundry cores, as proposed in the introduction. Now, grippers can be designed with a FEM simulation synchronously to the design of the core.

Based on the results in this article, we recommend using the weakest-link-based Mohr-Coulomb model for IOB core materials.

## 7. Conclusions

In this article, we introduced new and adapted known bi-axial testing methods to IOB core materials. We further showed, that inorganically-bound (IOB) core materials follow a Mohr-Coulomb failure criterion, when loaded with plane-stress states. A new failure criterion was proposed and validated, which combines weakest-link-theory and the Mohr-Coulomb criterion in one implicit equation. The failure model has only two parameters and can be parameterised with just a bending and a compression experiment in common sand laboratories. For the first time, a validated material model can be implemented in FEM calculations to predict the fracture of foundry cores under complex stress states. The testing methods in this article enable further research on the mechanical behaviour of core materials. For example, the failure surface of foundry cores after the casting is highly relevant for the decoring process. This failure surface could be characterised with the methodology introduced in this article. Furthermore, we will generalise the weakest-link-based Mohr-Coulomb failure criterion from plane-stress states to general stress states with tri-axial test methods in the upcoming month.

## Figures and Tables

**Table 1:** Data collection and statistical evaluation of the performed experiments.

Experiment	Stress Component	Sample Size	Scale Parameter	Shape Parameter	Mean	Standard Deviation
Uni-axial bending	$\sigma_{1}$	8	3.31 MPa	36	3.26 MPa	0.109 MPa
Uni-axial compression	$\sigma_{2}$	10	−8.01 MPa	11	−4.88 MPa	0.265 MPa
Bi-axial bending	$\sigma_{1}$/$\sigma_{2}$	12	2.69 MPa	19	2.05 MPa	0.130 MPa
Bi-axial compression	$\sigma_{2}$	23	−8.96 MPa	9.2	−5.81 MPa	0.674 MPa
Brazilian test	-	5	3.33 kN	14	3.21 kN	0.274 kN

## Data Availability

The data presented in this study are available on request from the corresponding author. The data are not publicly available since they are part of an ongoing study.

## Abbreviations

The following abbreviations are used in this manuscript:

3PB	three-point-bending
FEM	Finite element method
IOB	inorganically-bound
PTFE	Polytetrafluorethylen
PIA	principle of independent action
